# Polyphosphorus Ligand Complexes of Coinage Metals for Multiple Capturing of Intact P_4_ Tetrahedra

**DOI:** 10.1002/anie.202503614

**Published:** 2025-05-05

**Authors:** Claudia Göschl, Eugenia Peresypkina, Barbara Hiltl, Alexander V. Virovets, Gabor Balázs, Manfred Scheer

**Affiliations:** ^1^ Institute of Inorganic Chemistry University of Regensburg 93040 Regensburg Germany; ^2^ Institute of Inorganic and Analytical Chemistry Goethe‐University Frankfurt am Main Max‐von‐Laue‐Straße 7 60438 Frankfurt am Main Germany

**Keywords:** Coinage metal, Mixed‐ligand polyphosphorus complexes, Pentaphosphaferrocene, Supramolecular chemistry, White phosphorus

## Abstract

Two‐ and three‐component self‐assembly reactions of [Cp^R^Fe(η^5^‐P_5_)] (Cp^R^ = C_5_(CH_3_)_5_ (Cp*, **1a**), C_5_(4‐EtC_6_H_4_)_5_) (Cp^PEt^, **1b**)) with the coinage metal salts [Cu(CH_3_CN)_4_][SbF_6_] or AgSbF_6_ were investigated to study the prerequisites for the potential coordination of white phosphorus by using a sterically encumbered Cp^R^ ligand and noncoordinating anions. In the self‐assembly reactions with white phosphorus, either 0D or 1D coordination complexes are formed, all of which feature coordinated intact P_4_ tetrahedra and thus comprise an unprecedented class of mixed polyphosphorus ligand complexes capable of complete release of P_4_ in solution confirmed by NMR studies. The bulkiness of the used Cp^R^ ligand and the correctly chosen solvent allowed for obtaining more beneficial structural motifs, the first discrete tetra‐ and penta‐coordinated *cyclo*‐P_5_ ligand complexes, that provide the maximal content of coordinated P_4_ molecules per species known so far. All products are characterized by single‐crystal X‐ray diffraction, NMR spectroscopy, and mass spectrometry.

## Graphical abstract

Self‐assembly reactions of [Cp^R^Fe(η^5^‐P_5_)] (Cp^R^ = C_5_(CH_3_)_5_ and C_5_(4‐EtC_6_H_4_)_5_) with coinage metal salts and white phosphorus afforded an unprecedented novel class of mixed polyphosphorus ligand complexes in which metal ions multiply coordinate intact P_4_ tetrahedra. The bulkiness of the used Cp^R^ ligand and the right choice of the solvent tune the structure and the content of P_4_ in the coordination products that can release P_4_ upon decomplexation.

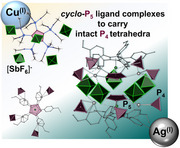

## Introduction

**Scheme 1:**
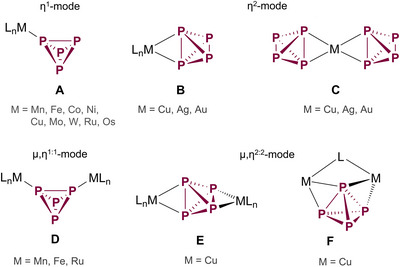
Different coordination types of intact P_4_ tetrahedra to metal centers.

White phosphorus (P_4_) is a demanded and demanding starting material as in industry as in academic research. In production, it is mainly used to obtain high‐purity organophosphorus and inorganic phosphorus compounds^[^ ^1^ ^]^ whereas research focuses on the activation of the P_4_ tetrahedron^[^ ^2^, ^3^, ^4^, ^5^, ^6^ ^]^ and thus the direct use of P_4_ for commercial products.^[^ ^7^, ^8^, ^9^, ^10^ ^]^ Due to the extreme reactivity of solid P_4_, both its stabilization and storage are of great importance. Certain progress in the stabilization of P_4_ molecules was made via their incorporation into porous materials,^[^ ^10^, ^11^, ^15^ ^]^ matrices of coordination polymers,^[^ ^13^ ^]^ cage compounds^[^ ^14^ ^]^ or discrete aggregates^[^ ^13^, ^14^ ^]^ that can be used to store and release P_4_ in subsequent reactions.^[^ ^11^, ^12^, ^13^, ^14^, ^15^ ^]^ These materials are either poorly soluble or often do not offer a convenient release of the captured P_4_ in an atom‐economical manner. All these approaches rest upon encapsulation^[^ ^11^, ^12^, ^13^, ^14^, ^15^ ^]^ that is less chemically advantageous than the coordination of the intact P_4_ molecules to transition metals^[^ ^16^, ^17^, ^18^, ^19^, ^20^, ^21^, ^22^, ^23^, ^24^, ^25^, ^26^, ^27^, ^28^ ^]^ provided that the resulting carrier complex for *n*(P_4_) is soluble and white phosphorus can be controllably released in full via complete decomplexation, e.g., by adding a convenient solvent. However, the coordination of the P_4_ tetrahedron, extensively studied in the context of the activation of white phosphorus, often leads to breaking P─P bonds.^[^ ^2^, ^3^, ^4^, ^5^, ^6^ ^]^ Only a handful of metal complexes with slightly depleted or intact P─P bonds have been reported since Sacconi et al. discovered the first complex [(np_3_)Ni(η^1^‐P_4_)] (np_3_ = tris(2‐diphenylphosphinoethyl)amine) containing an intact P_4_ tetrahedron in 1979.^[^ ^16^ ^]^ Besides η^1^‐(**A**)^[^ ^16^, ^17^, ^18^ ^]^ or η^2^‐coordination (**B** and **C**),^[^ ^17^, ^18^, ^19^, ^20^, ^21^, ^22^, ^23^, ^24^, ^25^ ^]^ P_4_ can also bridge two metal centers (Scheme 1). The metals can bind two P atoms in a μ,η^1:1^‐fashion (**D**)^[^ ^19^, ^23^, ^25^, ^26^ ^]^ or two opposing (**E**)^[^ ^22^, ^24^, ^28^ ^]^ as well as adjacent (**F**)^[^ ^27^ ^]^ edges of the P_4_ tetrahedron.

Notably, for the complex [(LCu)_2_(μ,η^2:2^‐P_4_)] (L = [{N(C_6_H_3_ *i*Pr_2_‐2,6)C(Me)}_2_CH]^−^), a release of intact P_4_ molecules was observed; [Ag(η^2^‐P_4_)_2_][Al(OR_F_)_4_] (R_F_ = OC(CF_3_)_3_) is also reported as a suitable agent for the release of intact P_4_ molecules under special reaction conditions.^[^ ^24^, ^27^ ^]^ Analyzing these data, three crucial drawbacks are to be overcome: 1) Only one or two P_4_ tetrahedra are bound in a discrete complex. Loading a complex with multiple P_4_ molecules would be desirable and could be enabled by using polynuclear transition metal complexes. 2) The coordination of P_4_ molecules must not compete with other ligands or counter anions as shown by complex **C** with weakly coordinating anions (WCAs), which assist in the coordination of two P_4_ tetrahedra. 3) An appropriate type of metal complexes to serve as an efficient carrier of P_4_ is still to be found.

**Scheme 2:**
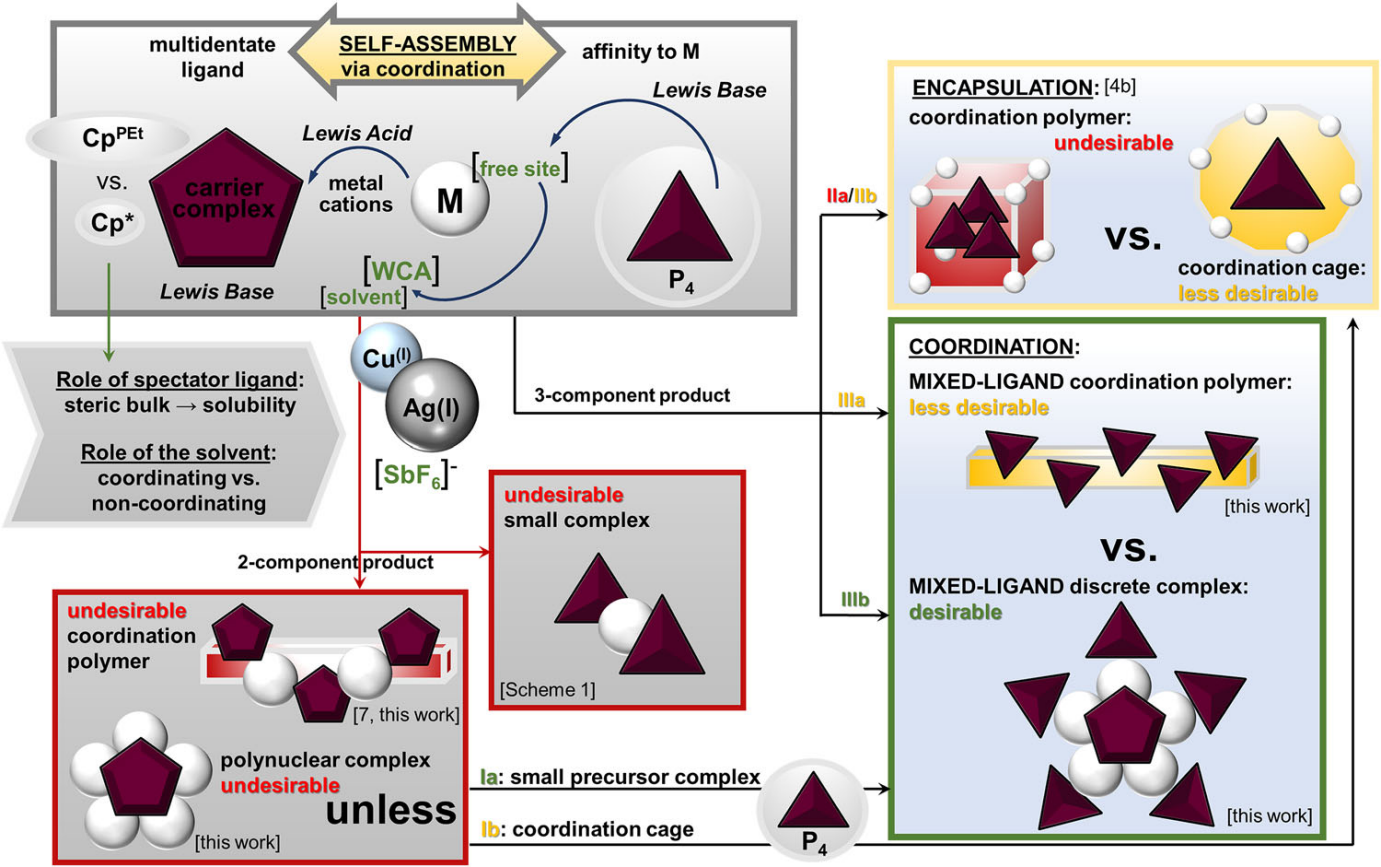
Possible strategies to immobilize intact P_4_ tetrahedra.

Specific chemical environment supporting the realization of the coordination scenario (not excluding, however, less desirable encapsulation of P_4_) can be provided by self‐assembly systems containing pentaphosphaferrocene [Cp^R^Fe(η^5^‐P_5_)] (**1**, Cp^R^: Cp* = C_5_(CH_3_)_5_ (**1a**), Cp^Bn^ = C_5_(CH_2_Ph)_5_, Cp^BIG^ = C_5_(4‐*n*BuC_6_H_4_)_5_)^[^ ^29^, ^30^, ^31^ ^]^ and coinage metal cations. The polydentate *cyclo*‐P_5_ ligand of **1** can coordinate transition metal centers adopting a full row from mono‐ to penta‐coordination.^[^ ^32^ ^]^ This has made the *cyclo*‐P_5_ ligand complexes outstanding building blocks and gave rise to a variety of coordination polymers and discrete coordination cages over the past years.^[^ ^32^, ^33^, ^34^, ^35^, ^36^, ^37^, ^38^, ^39^ ^]^ It was even possible to incorporate various guest molecules^[^ ^33^, ^34^, ^35^, ^36^, ^37^ ^]^ into both the polymeric matrices and giant cages of **1a** and CuX (X = Cl, Br, and I), including, among others, the P_4_ and As_4_ tetrahedral molecules (Scheme 2).^[^ ^12^ ^]^ Building a discrete transition metal complex on the pentaphosphaferrocene platform could provide a suitable chemical and coordination environment for the targeted purpose.

In our endeavor to develop the coordination chemistry of **1** and facilitate three‐component self‐assembly reactions with this building block, an approach to use coinage metal salts of WCAs proved successful.^[^ ^40^, ^41^, ^42^, ^43^, ^44^, ^45^, ^46^, ^47^ ^]^ Noncoordinating behavior of WCAs leaves coordination sites at the coinage metal cation available for additional coordination.^[^ ^43^, ^44^, ^45^ ^]^ This approach allowed not only to circumvent the insolubility of the Ag halides and involve Ag^+^ in the self‐assembly reactions but also to use a series of organic dinitriles to access three‐component coordination polymers as well as discrete supramolecular species lined in coordination networks.^[^ ^44^ ^]^ With this in mind and due to the proven ability of WCAs to stabilize charged species and weakly bound complexes,^[^ ^48^, ^49^ ^]^ we considered multiple coordination of the very weak donor P_4_ feasible, although no examples of mixed‐ligand polyphosphorus complexes have been reported so far, except for [Co(η^5^‐P_5_){η^2^‐P_2_H(Mes)}]^2−^ obtained by chemical activation of the Zintl phase P_7_^3−^ and possessing an organo‐substituted phosphorus ligand in addition to the *cyclo*‐P_5_ ligand.^[^ ^50^ ^]^

Based on our wide‐ranging experience, the self‐assembly system of **1**, WCA salts of coinage metal cations and P_4_ can give different outcomes **I**–**III** (Scheme 2): Similar to the reactions of P_4_ with Ag[Al(OR_F_)_4_] (OR_F_ = OC(CF_3_)_3_, OC(CH_3_)(CF_3_)_2_),^[^ ^20^ ^]^ it can facilitate the coordination of the P_4_ tetrahedron as a weak ligand within discrete or polymeric complexes (**IIIa**,**b**), or P_4_ is to be encapsulated as a guest in a hosting cage formed by *cyclo*‐P_5_ ligand complexes and metal ions similar to other host–guest compounds based on pentaphosphaferrocene (**Ib**, **II**).^[^ ^12^, ^32^ ^]^ Both types of resulting products could be applied either as a storage material for P_4_ or as starting material for further reactions involving white phosphorus. However, the most beneficial outcome of the self‐assembly would be a compact, soluble, discrete complex of **1** bearing multiple metal centers loaded with intact P_4_ molecules (**Ia**). However, known molecular or cationic complexes of **1** are scarce and do not comply with these requirements.^[^ ^32^ ^]^

Within our concept, different factors can be varied to tune the self‐assembly: 1) The Lewis acidity of the metal cation can change the type of the resulting metal complex. Harder Cu^+^ or softer Ag^+^ should rather coordinate the *cyclo*‐P_5_ ligand or P_4_, respectively, that can switch the self‐assembly from molecular complexes or coordination cages, i.e., between coordination versus encapsulation scenarios. 2) Hexafluoroantimonates [Cu(CH_3_CN)_4_][SbF_6_] or AgSbF_6_ were chosen because an [SbF_6_]^−^ anion can either act as a WCA or complete, if necessary, the coordination sphere of the metal ion by weak coordination preventing undesirable condensation to a polymeric product. 3) Based on previous experience, the solubility of pentaphosphaferrocene complexes can be tuned by the specific Cp^R^ ligand,^[^ ^33^, ^34^, ^35^, ^36^, ^37^ ^]^ therefore, using **1a** and the bulkier [Cp^PEt^Fe(η^5^‐P_5_)] (Cp^PEt^ = C_5_(4‐EtC_6_H_4_)_5_, **1b**)^[^ ^30^ ^]^ can reveal the role of this factor as well as of the steric bulk of Cp^R^ on the resulting polynuclear complex. Given numerous self‐assembly routes, we considered it necessary first to explore the two‐component self‐assembly of **1a** and **1b** with [Cu(CH_3_CN)_4_][SbF_6_] or AgSbF_6_ depending on the stoichiometry and used solvent (Scheme 2). Afterward, three‐component self‐assembly reactions with white phosphorus were performed, resulting in first compounds containing two different polyphosphorus ligands, among them an unprecedented molecular complex in which a *cyclo*‐P_5_ ligand coordinates four very weakly bound intact P_4_ units.

## Results and Discussion

### Preliminary Studies: Two‐Component Self‐Assembly of 1 and [Cu(CH_3_CN)_4_][SbF_6_]

**Scheme 3:**
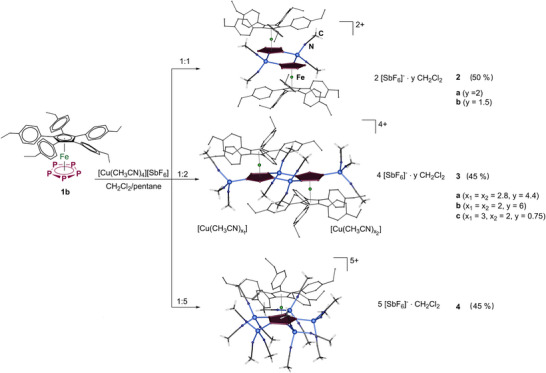
Self‐assembly reactions of **1b** with [Cu(CH_3_CN)_4_][SbF_6_] resulting in discrete complexes **2**–**4**. Yields are given in parentheses.

Two‐component self‐assembly of **1a** and similar [Cu(CH_3_CN)_4_][WCA] (WCA = BF_4_^−^, PF_6_^−^) has been studied previously.^[^ ^32^, ^47^, ^51^ ^]^ In the reaction of **1b** with [Cu(CH_3_CN)_4_][SbF_6_] different coordination products crystallize in moderate yields upon variation of the stoichiometry of the reactants (Scheme 3). After stirring **1b** and corresponding amounts of [Cu(CH_3_CN)_4_][SbF_6_] in CH_2_Cl_2_ for 20 min, the brownish solutions are filtered into a Schlenk tube. The crystalline products **2**–**4** can be isolated by careful layering of the solutions with *n*‐pentane (Scheme 3).

According to X‐ray crystallography (SC‐XRD),^[^ ^52^ ^]^ the dimeric cationic complexes of two triclinic phases [(**1b**)Cu(CH_3_CN)_2_]_2_[SbF_6_]_2_ · *y* CH_2_Cl_2_ (bright green plates, **2a**: *y* = 2, **2b**: *y* = 1.5) consists of two units **1b** bridged by two Cu^+^ cations. Each unit **1b** is coordinated by Cu^+^ cations in a 1,2‐coordination mode, forming a six‐membered {Cu_2_P_4_} ring (Scheme 3). Each tetrahedral Cu^+^ cation is coordinatively saturated by two acetonitrile ligands. During sample handling, large green prisms of **3** were noticed to rapidly recrystallize in the mother solution. Hereby, the formation of at least three crystalline phases [(**1b**)_2_Cu_4_(CH_3_CN)_4+_ *_x_*][SbF_6_]_4_ · *y* CH_2_Cl_2_ (**3a**: *x* = 5.6, *y* = 4.4; **3b**: *x* = 4, *y* = 6; **3c**: *x* = 5, *y* = 1) was identified by SC‐XRD, all differing in the content of coordinated CH_3_CN and CH_2_Cl_2_ solvent molecules. All complexes **3** feature similar dimeric cations as in **2**; however, they contain two additional Cu^+^ cations resulting in a 1,2,4‐coordination mode of each *cyclo*‐P_5_ ligand (Scheme 3). The different coordination geometries of copper(I), trigonal or tetrahedral, and therefore the various contents of acetonitrile ligands, highlight the differences in the compositions and molecular structures of the crystalline phases **3a**–**3c** (see Supporting Information).

A more interesting structural motif is formed when using 5 equiv of [Cu(CH_3_CN)_4_][SbF_6_] in the reaction with **1b** (Scheme 3), which results in the formation of triclinic khaki‐green complex [(**1b**)Cu_5_(CH_3_CN)_15_][SbF_6_]_5 _· CH_2_Cl_2_ (**4**). Every P atom of the *cyclo*‐P_5_ ligand of **1b** is coordinated to a Cu^+^ cation whose tetrahedral environment is completed by three CH_3_CN ligands. As a result, the first discrete monomeric structure of a penta‐coordinated *cyclo*‐P_5_ building block is formed, bearing five additional Cu^+^ centers, potentially for multiple coordination of P_4_. Complex **4** is well‐soluble and structurally beneficial precursor for this attempt.

Compounds **2**, **3**, and **4** are very well soluble in CH_2_Cl_2_ enabling their investigation by NMR spectroscopy in solution. The ^1^H NMR spectra of **2** and **4** show the amount of CH_3_CN that agrees with the X‐ray data; the spectrum of **3** reveals an average number of 8.7 CH_3_CN that is also confirmed by the elemental analysis. The ^31^P{^1^H} NMR spectra show a correlation between the number of coordinated Cu^+^ cations in **2**–**4** and the shift of the signal of the P atoms of the *cyclo*‐P_5_ ligand: The ^31^P{^1^H} NMR signal for **2** with a 1:1 ratio (**1b**:[Cu(CH_3_CN)_4_][SbF_6_]) is detected at 140.4 ppm (rotation of the *cyclo*‐P_5_ ligand) and is considerably downfield shifted compared to the resonance signal found for free **1b** (172 ppm). When increasing the content of Cu(I) salt in compounds **3** and **4** to 1:2 and 1:5 ratios, the signal of the products shifts further downfield to 125.1 and 108.2 ppm, respectively. In each of the ESI MS spectra of compounds **2–4**, the highest peak at *m*/*z* = 900.11 was attributed to [(**1b**)Cu(CH_3_CN)]^+^. The base peak at *m*/*z* = 144 corresponds to [Cu(CH_3_CN)_2_]^+^.

*Using small precursor complexes* ***2***–***4*** *for the P_4_ capture*. The complexes **2**–**4** with labile CH_3_CN ligands in the coordination sphere of copper were attempted as small precursor complexes for white phosphorus capture (Scheme 2, **Ia**). These attempts have failed most probably due to the lower donor ability of P_4_ in comparison to CH_3_CN. An attempt to avoid this competition by starting from [Cu(mesitylene)_2_][SbF_6_]^[^ ^53^ ^]^ salt only led to the formation of poor‐quality crystalline material and/or oily residues. Given these drawbacks, it seemed reasonable to abstain from using [Cu(CH_3_CN)_4_][SbF_6_] and turn to AgSbF_6_ instead, which does not necessarily need a stabilizing environment of coordinated solvents like CH_3_CN. It was recently shown that in the self‐assembly of **1a** and AgSbF_6_, the Ag^+^ cations provide additional coordination sites that can be occupied by flexible aliphatic as well as rigid aromatic dinitriles as a third component.^[^ ^44^, ^45^ ^]^ This encouraged us to introduce such a weak donor as white phosphorus in similar reactions after studying first corresponding two‐component self‐assembly systems to make sure that Ag(I) can form similar complexes with molecular structures supportive of multiple coordination of small molecules compared to copper.

### Preliminary Studies: Two‐Component Self‐Assembly of [(Cp*Fe(η^5^‐P_5_)] (1a) and AgSbF_6_ in Noncoordinating Solvents

**Scheme 4:**
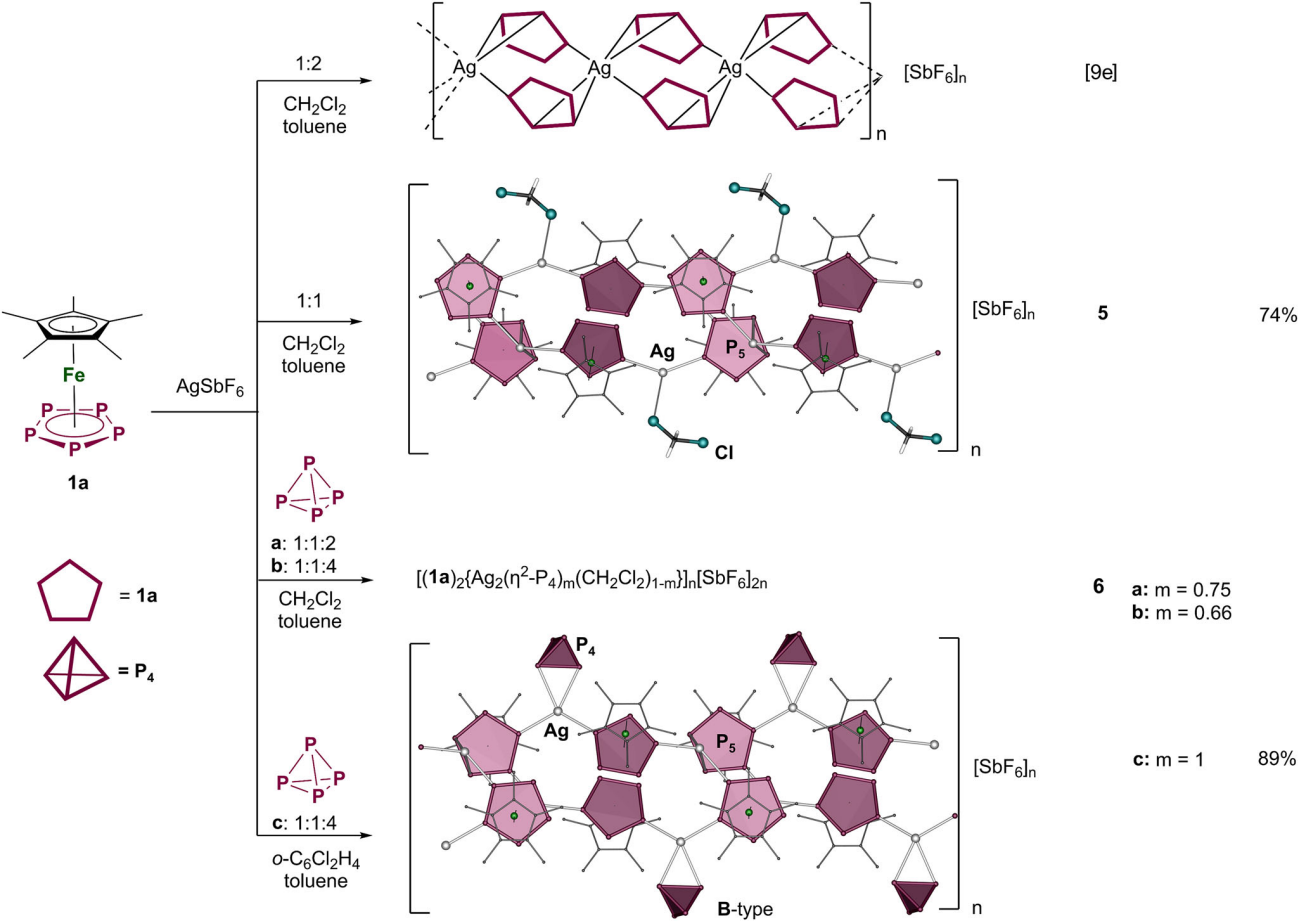
Self‐assembly reaction of **1a** with AgSbF_6_. Yields are given in parentheses.

In the two‐component self‐assembly reaction of AgSbF_6_ and **1a**, only a 1D polymer [(**1a**)_2_Ag]_n_[SbF_6_]_n_ has been isolated so far (Scheme 4).^[^ ^44^ ^]^ When reducing the ratio **1a**:AgSbF_6_ of 1:2 to 1:1 ratio, another 1D polymer [(**1a**)_2_{Ag_2_(CH_2_Cl_2_)}]*_n_* [SbF_6_]_2_ *_n_* (**5**) is observed to crystallize in the triclinic crystal system. The repeating unit of this double‐stranded polymer is built up by two moieties **1a** and two Ag^+^ cations (Scheme 4). Within this unit, one *cyclo*‐P_5_ ligand is coordinated to Ag^+^ cations in a 1,3‐mode, whereas the second moiety **1a**, displays a side‐on and end‐on coordination of three P atoms to two Ag^+^ cations. Additionally, the side‐on coordinated Ag^+^ cation bridges two strands of **5** via additional end‐on coordination of a P atom of an adjacent moiety **1a**. The Ag^+^ cations weakly coordinate either [SbF_6_]^−^ anions or CH_2_Cl_2_ molecules displaying a pseudo‐tetrahedral or trigonal environment.^[^ ^54^, ^55^, ^56^ ^]^ Crystals of **5** are insoluble in common solvents such as *n*‐hexane, toluene, or CH_2_Cl_2_. The addition of CH_3_CN or pyridine to a suspension of **5** in CH_2_Cl_2_ gives an orange solution due to readily undergoing fragmentation. For the NMR spectroscopic investigations, the solvent was removed, and the residue was redissolved in CD_2_Cl_2_. In the ^1^H NMR and ^31^P{^1^H} NMR spectra of solution of **5** in CD_2_Cl_2_ prepared in this way, only signals corresponding to the free starting material are detected. In the ^1^H NMR spectrum of **5** in CD_3_CN, the signals of **1a** and CH_2_Cl_2_ present in the crystal lattice are visible in the expected ratio of 2:1. The ^31^P{^1^H} NMR spectrum shows only a broad signal at 138 ppm corresponding to **1a** coordinated to the Ag^+^ cations in a dynamic equilibrium. In the ESI MS spectrum of **5** in CH_3_CN/CH_2_Cl_2_, peaks for oligomeric fragments are detected. The peak with the highest *m*/*z* at 2521.98 can be attributed to [(**1a**)_4_Ag_4_(SbF_6_)_3_]^+^.

Formation of **5** did not provide an optimal precursor complex, but the fact that the silver cations in **5** coordinate either weakly bound [SbF_6_]^−^ anions or CH_2_Cl_2_ molecules, the coordination of P_4_ molecules also seems feasible in this system.

### Three‐Component Self‐Assembly of [(Cp*Fe(η^5^‐P_5_)] (1a) with AgSbF_6_ and White Phosphorus

By layering AgSbF_6_ in CH_2_Cl_2_ or *ortho*‐dichlorobenzene with a mixture of **1a** and P_4_ in toluene in different stoichiometric ratios, crystals of [(**1a**)_2_{Ag_2_(η^2^‐P_4_)*_m_* (CH_2_Cl_2_)_1‐_ *_m_*}]*_n_* [SbF_6_]_2_ *_n_* (**6**; *m* = 0.75 (**6a**), 0.67 (**6b**), 1 (**6c**)) are isolated in good yields (Scheme 4). All three products are isostructural and possess similar double‐stranded structures as in **5**, with the difference that the Ag^+^ cations corresponding to those that coordinate CH_2_Cl_2_ in **5** partly coordinate either a CH_2_Cl_2_ molecule or a P_4_ tetrahedron, acting as η^2^‐coordinating ligands. The P─P bond lengths in the coordinated P_4_ tetrahedra in **6c** are in agreement with that reported in literature for an intact P_4_ tetrahedron coordinated in an η^2^‐mode to a coinage metal cation (Table 1).^[^ ^20^, ^21^, ^22^, ^24^, ^57^, ^58^ ^]^ The P─P bond distances for adjacent and opposite to the coordinated bonds corroborate those found in the intact P_4_ molecule (2.1994(3)–2.2228(5) Å).^[^ ^57^, ^58^, ^59^, ^60^ ^]^ The coordinated ones are slightly elongated. The single‐point DFT computations using the experimental geometry of **6c** at the D3^[^ ^61^ ^]^‐B3LYP^[^ ^68^, ^69^ ^]^/def2‐TZVPP^[^ ^68^, ^69^ ^]^ level of theory further confirm an elongated but intact nature of the coordinated P─P bond (see Supporting Information). The content of the coordinated P_4_ depends on the relative amount of white phosphorus as well as on the solvents used (Scheme 4). In **6a**, a 1:1:2 ratio (**1a**:AgSbF_6_:P_4_) in CH_2_Cl_2_ leads to P_4_ ligands and CH_2_Cl_2_ molecules sharing the same crystallographic position with an 0.75/0.25 occupancy. Counterintuitively, introducing a fourfold excess of white phosphorus in **6b** in CH_2_Cl_2_ slightly decreases the occupancy of P_4_ to 67 %, which is why the pure P_4_‐containing compound **6c** cannot be obtained in a straightforward way. To avoid competitive coordination of CH_2_Cl_2_ and P_4_, *ortho*‐dichlorobenzene was used instead as a noncoordinating solvent for AgSbF_6_. In this way, **6c** containing a maximum amount of P_4_ was obtained selectively (Scheme 4). Surprisingly, **6a** can be stored as a crystalline solid at room temperature in air for weeks without decomposition as confirmed by SC‐XRD (see Supporting Information), whereas **6c** has decomposed in air after 3 days. Therefore, a moderate content (25%–30%) of CH_2_Cl_2_ in the positions of P_4_ seems to stabilize **6**.

**Table 1:** Selected bond lengths and angles for coordination products of P_4_ and coinage metal cations.^a)^

Compound	M–P(P_5_)	M–(sp^2^–C)	P─P(P_5_)	M─P(P_4_)	(P─P)_coord_ ^b)^	(P─P)_adj_	(P─P)_opposite_	P(P_4_)–M–(P_4_)
**6c**	2.4606(17); 2.673(2)–2.678(2)^c)^	–	2.106(3)–2.152(3)	2.678(2)–2.700(2)	2.297(3)	2.153(3)–2.172(3)	2.191(4)	50.57(6)
**11**	2.5051(8)–2.5289(7)	2.618(3)– 2.661(3)	2.1098(11)–2.1135(11)	2.5825(9)–2.6603(9)	2.302(1)–2.326(1)	2.156(2)–2.183(1)	2.205(2)–2.210(2)	52.07(3)–53.07(3)
**12a**	2.4938(15)–2.5233(15)	2.598(6)–2.661(5)	2.101(2)–2.167(3)	2.569(2)–2.655(2)	2.263(3)–2.310(2)	2.154(3)–2.176(3)	2.147(4)–2.200(3)	47.81(13)–52.73(6)
**12b**	2.4939(14)–2.664(8)	2.114(2)–2.700(10)	2.107(2)–2.114(2)	2.53(2)–2.75(2)	2.266(3)–2.348(9)	2.020(2)–2.248(14)	2.152(7)–2.200(6)	50.4(5)–54.9(5)
[LCu(η^2^‐P_4_)]^d)^	–	–	–	2.2592(6)–2.2707(6)	2.386(4)	2.180(4)–2.280(4)	2.141(6)	63.10(12)
[Ag(η^2^‐P_4_)][Al(OR_F_)_4_]^e)^	–	–	–	2.5262(8)–2.5274(9)	2.308(1)	2.152(1)–2.174(1)	2.188(2)	54.34(3)
[lPrAuP_4_][SbF_6_]^f)^	–	–	–	2.4333(7)–2.4352(6)	2.357(1)	2.164(1)–2.170(1)	2.194(1)	57.79(7)

^a)^ In Å and degrees, respectively. In **6c**‐**12,** M = Ag. The distances with low occupied atoms are not listed (see Supporting Information for details).

^b)^ Bond lengths in the P_4_ tetrahedra are given as (P─P)_adj._ and (P─P)_opposite_ for adjacent and opposite bonds with respect to the coordinated (P─P)_coord._ bond.

^c)^ Intervals for end‐on and side‐on coordination are shown.

^d)^ L is the β‐diketiminato ligand [{N(C_6_H_3_ *i*Pr_2_‐2,6)C(Me)}_2_CH]^−^.^[^ ^24^ ^]^

^e)^ OR_F_ = OC(CH_3_)(CF_3_)_2_.^[^ ^20^ ^]^

^f)^ lPr = 1,3‐bis(2,6‐diisopropylphenyl)‐1,3‐dihydro‐2*H*‐imidaz‐ol‐2‐ylidene reported in Ref. [57].

**Figure 1:**
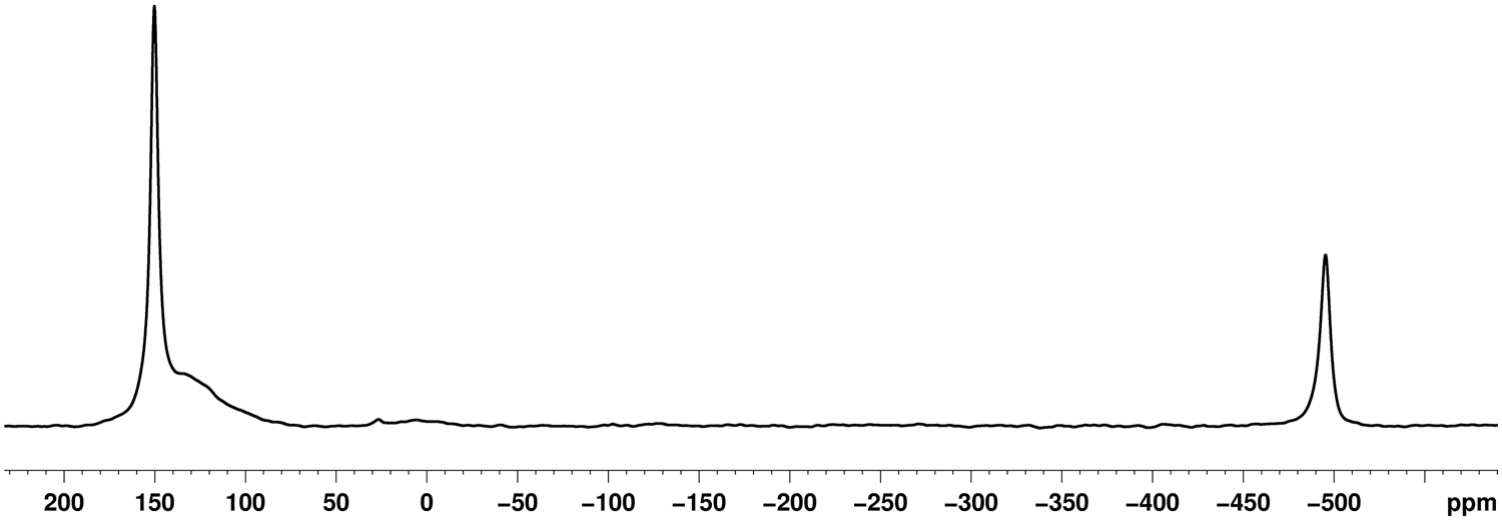
^31^P{^1^H} MAS NMR spectrum of **6c**.

The ^31^P{^1^H} MAS NMR spectrum of crystalline **6** confirms coordination of the P_4_ and agrees with the SC‐XRD data (Figure 1): A sharp signal at 150.2 ppm and a small broad signal at 133.4 ppm are detected, which can most probably be attributed to the different coordination modes of the P atoms of the *cyclo*‐P_5_ units. Additionally, a singlet at −495.4 ppm is observed, which can be assigned to the coordinated intact P_4_ tetrahedra, which is in agreement with the value of −497 ppm reported for [Ag(η^2^‐P_4_)_2_][Al(O*^i^* Pr_F_)_4_]).^[^ ^20^ ^]^ The Raman spectrum of solid **6** reveals bands similar to those found for free **1a** and for solid P_4_, respectively (Table S1 and Figure S8). In the ESI MS spectrum of **6** in CH_3_CN/CH_2_Cl_2_, the peak with the highest mass‐to‐charge ratio can be attributed to [(**1a**)_4_Ag_4_(SbF_6_)_3_]^+^ at *m*/*z* = 2521.98. White phosphorus is observed to get released from the polymer by grinding the crystals of **6** and readily reacts with oxygen when exposed to air.

*Using mixed‐ligand polymer* ***6*** *for the P_4_ release*. Stable in air, crystalline **6** is insoluble in common solvents such as toluene or CH_2_Cl_2_ but can be dissolved in donor solvents under partial fragmentation of the polymeric structure, suggesting a convenient release of the coordinated P_4_. To confirm this by NMR investigations in solution, crystals of **6** were directly dissolved in CD_3_CN. In this case, a weak singlet at −525 ppm can be observed in the ^31^P{^1^H} NMR spectrum, which is attributed to free P_4_, in addition to a similar signal for **1a** at 136 ppm. When CH_3_CN or pyridine was added to a suspension of **6** in CH_2_Cl_2_, the solvent mixture was removed, and the residue was redissolved in CD_2_Cl_2_. In the ^1^H NMR and the ^31^P NMR spectra in CD_2_Cl_2_ of such solutions of **6**, only signals of free **1a** and P_4_ are detected, confirming complete release of coordinated molecules.

Despite reaction of **1a** with AgSbF_6_ and P_4_ following the less desirable route **IIIa** (Scheme 2) toward complex **6** with relatively low content of P_4_, novel mixed‐ligand complexes that can coordinate multiple P_4_ tetrahedra are obtained. However, further improvements can be made when using the starting complex **1b** instead of **1a** and avoiding competition between P_4_ and weakly coordinating solvent.

### Two‐Component Self‐Assembly of [(Cp^PEt^Fe(η^5^‐P_5_)] (1b) and AgSbF_6_ in Noncoordinating Solvents

**Scheme 5:**
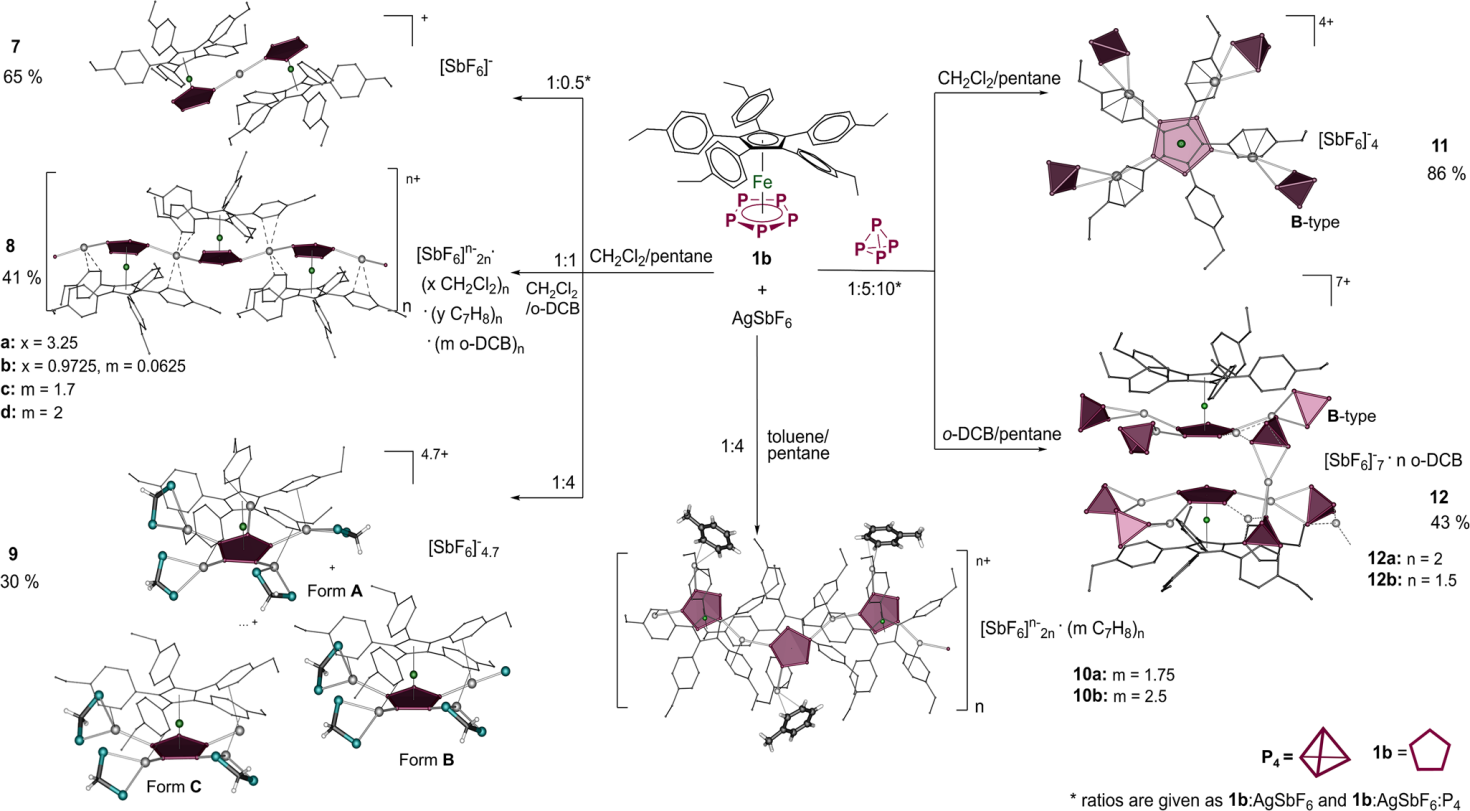
Two‐ and three‐component self‐assembly reactions of **1b** with AgSbF_6_ and white phosphorus. Yields are given in parentheses.

Having succeeded in the synthesis of mixed P_4_/P_5_‐ligand complexes of silver with **1a**, it was necessary to improve the solubility by introducing **1b** bearing the bulkier Cp^PEt^ ligand that also simultaneously changes the steric demand of the Cp^R^ spectator ligand at **1**. The influence of this factor on the reaction pathway and whether the prerequisites for the coordination of P_4_ molecules still apply are the incentives for this section. The two‐component self‐assembly reaction of AgSbF_6_ with **1b** afforded various products depending on the stoichiometry used (Scheme 5). According to the general procedure used for all these reactions, after stirring both components in CH_2_Cl_2_ for at least 30 min, the solution was filtered and layered with pentane or *o*‐C_6_H_4_Cl_2_, which led to the formation of crystalline products in moderate yields.

The SC‐XRD^[^ ^52^ ^]^ of **7** reveals a discrete dimeric complex [(**1b**)_2_Ag][SbF_6_], in which two *cyclo*‐P_5_ ligands are bridged by an Ag^+^ cation. The two different monoclinic crystalline phases of [(**1b**)Ag]*_n_* (SbF_6_)*_n_* (**8a** and **8b**) form with an equimolar stoichiometry. SC‐XRD shows that in the 1D polymer **8a**, *cyclo*‐P_5_ ligands are 1,3‐coordinated to two Ag^+^ cations each and the Ag^+^ cations coordinate two molecules of **1b**. The distorted tetrahedral environment of Ag^+^ is completed by π‐interaction with the phenyl groups of the Cp^PEt^ ligands so that Ag^+^ cations slightly deviate from the *cyclo*‐P_5_ planes.

Compound **8b** contains two isomeric entities: One entity and a major part of the disordered second one correspond to a 1D polymeric chain as in **8a**; the minor part (0.1) represents an overlay of discrete complexes [(**1b**)Ag]^+^ formed with different positions of disordered Ag cations (see Supporting Information for detail). The connectivity of the cationic part of **8a** is comparable to the structure of [{Cp^BIG^Fe(η^5^‐P_5_)}Ag]*_n_* [Al(OC(CF_3_))_4_]*_n_* that also contains a bulkier pentaphosphaferrocene and a weakly coordinating anion.^[^ ^41^, ^48^ ^]^ The reactions of the less sterically demanding **1a** with Ag[Al(C(CF_3_)_3_)_4_] or AgSbF_6_ both lead to 1D cationic structures in which the Ag(I) coordination sphere is saturated not by π‐interaction with bulky Cp^PEt^ ligands but by the P_5_ ligands of two additional units of **1a**.^[^ ^40^, ^44^ ^]^ Therefore, the bulkiness of the Cp^R^ ligand is more of an overwhelming factor for the nature of the resulting product than the steric demand of the anion. This factor can be used to tune the solubility of the products without impeding multiple coordination of silver cations.

When using *ortho*‐dichlorobenzene as solvent instead of CH_2_Cl_2_ in the reaction of **1b** with AgSbF_6_ in a ratio of 1:1, two different crystalline phases were isolated revealing the 1D polymers [(**1b**)Ag]*_n_* [SbF_6_]*_n_* ·*m* *o*DCB (*m* = 1.7*n* (**8c**), 2*n* (**8d**)) with similar structural motifs as observed for **8a** but with a different packing of the chains (see Figure S29). Due to no change in the structural behavior, no further investigations were attempted in this media.

**Figure 2:**
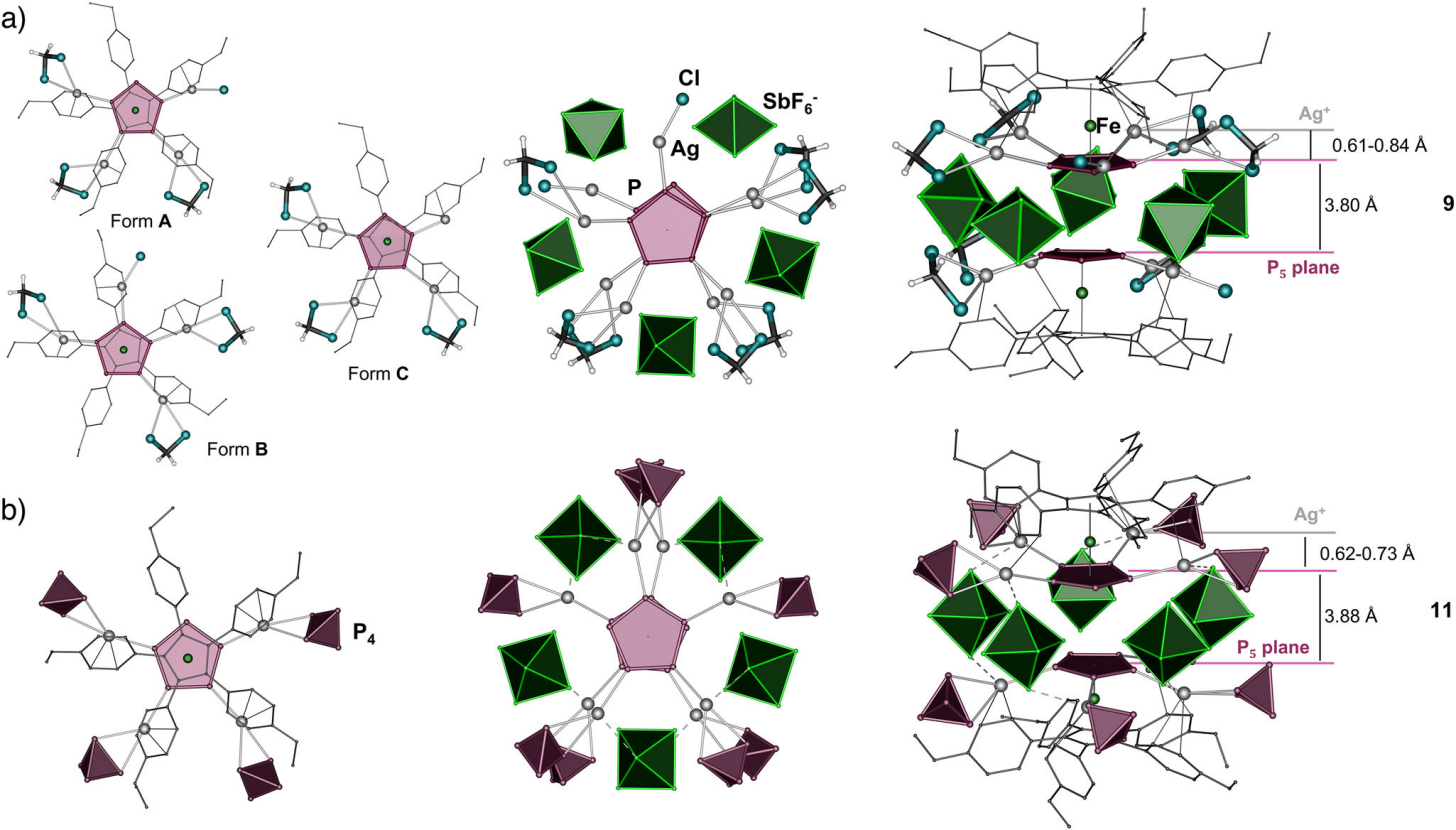
Comparison of complexes a) **9** (major forms) and b) **11**. [SbF_6_]^−^ are shown as octahedra; disorder and outer sphere [SbF_6_]^−^ and CH_2_Cl_2_ are not shown.

Increasing the relative amount of AgSbF_6_ to 4 equiv (Scheme 5) leads to the discrete complex [(**1b**)_2_Ag_7.5_Cl_0.6_(CH_2_Cl_2_)_5.7_][SbF_6_]_6.9_ · 2 CH_2_Cl_2_ (**9**) crystallizing in the monoclinic space group *P*2_1_/*n*. The SC‐XRD shows that **9** contains two opposing units of **1b** with two slightly off‐set and almost parallel *cyclo*‐P_5_ ligands suggesting a certain stabilization by weak π–π interactions (Figure 2). The overall composition of **9** amounts to a noninteger number (7.5) of AgSbF_6_ per formula unit implying that **9** consists of structurally similar complexes of different composition that cocrystallize in the solid state (Scheme 5). Formally, the average amount of the Ag^+^ cations coordinated to one moiety of **1b** and disordered over four positions is 3.95, reflecting a mixture of tri‐ and tetra‐coordinated *cyclo*‐P_5_ ligands in a ratio of 0.05:0.95. The other moiety of **1b** is coordinated by an average amount of 3.55 Ag^+^ ions at all five possible positions allowing penta‐, tetra‐, and mono‐coordinated complexes of Ag(I) (Figure S31). Due to this disordering, the structure of individual cations cannot be unambiguously derived. However, the structural motif of **9** seems beneficial for making good carrier complex for multiple coordination of P_4_ molecules.

Interestingly, two of the Ag^+^ cations in **9** (Figure 2a) are coordinated by Cl^−^ anions (Table S30) formed most likely by abstraction of Cl^−^ from dichloromethane.^[^ ^70^, ^71^, ^72^, ^73^ ^]^ These partly occupied Cl^−^ anions balance the positive charge in **9**. Several SC‐XRD experiments show that a similar content of the coordinated Cl^−^ is always present in different crystalline samples. The remaining Ag^+^ cations coordinate CH_2_Cl_2_ molecules in an asymmetric η^2^‐mode. The coordination sphere of the Ag^+^ cations is completed by π‐interactions via aromatic bonds of the Cp^PEt^ ligand, which causes a deviation of the Ag^+^ cations from the *cyclo*‐P_5_ plane. Furthermore, 4.85 [SbF_6_]^−^ anions are distributed over five positions in the cavity between the Ag^+^ cations of the {(**1b**)Ag*_x_*} fragments (Figures 2a and S32); two of the [SbF_6_]^−^ anions are terminally coordinated to Ag^+^ cations and another two bridge Ag^+^ cations of different complexes. An excess of AgSbF_6_ in the reaction allows neither to saturate partly occupied positions of Ag^+^ nor to change the composition of **9**.

Crystals of **7**, **8a**, and **9** are poorly soluble in CH_2_Cl_2_ but dissolve in a mixture of CH_2_Cl_2_ and CH_3_CN undergoing partial fragmentation. As a result, the ^31^P{^1^H} NMR spectra of **7** and **8a** in CD_2_Cl_2_/CD_3_CN show sharp singlets at 161 and 156 ppm, respectively, which are highfield shifted compared to the signal typical of the free pentaphosphaferrocene **1b** (172 ppm), whereas the observed singlet at 152.2 ppm for **9** is slightly downfield shifted. This fragmentation can also be detected in the ESI MS spectra of compounds **7**–**9**, all of them with the highest peak at *m*/*z* = 905.06, which can be attributed to a [(**1b**)Ag]^+^ moiety.

Using toluene instead of CH_2_Cl_2_ in a similar reaction, crystal formation can be observed after layering the reaction mixture of **1b** and AgSbF_6_ (ratio 1:4) with *n*‐pentane. SC‐XRD reveals another 1D polymer with the general formula [(**1b**)Ag_2_]*_n_* [SbF_6_]_2_ *_n_* · *m* C_7_H_8_ (*m* = 1.75*n* (**10a**), 2.5*n* (**10b**)). However, the synthesis of **10** is accompanied by the formation of unavoidable oily residues as well as by undergoing redox processes making the isolation of analytically pure compounds in good yield problematic (see Supporting Information for detail).

Due to the weak coordination of the CH_2_Cl_2_ molecules exemplified by compounds **5** and **9** as well as the π‐coordination to the aromatic moieties of the Cp^PEt^ ligands, the prerequisites for the coordination of white phosphorus to Ag(I) seem feasible under similar conditions (Scheme 2). Complex **5** exemplifies the less desirable route **IIIa** to give a polymeric matrix for P_4_ coordination, whereas complex **9** resembling copper complex **4**, promises to be an effective carrier for higher P_4_ load.

### Three‐Component Self‐Assembly of [(Cp^PEt^Fe(η^5^‐P_5_)] (1b) with AgSbF_6_ and White Phosphorus

To introduce P_4_ into the self‐assembly of **1b** and AgSbF_6_, the pentaphosphaferrocene **1b**, AgSbF_6_, and P_4_ in a 1:5:10 ratio are stirred together in CH_2_Cl_2_ for 1 h at most. The turbid brown solution is filtered and layered with *n*‐pentane. After complete diffusion, brown prisms of the monomeric complex [(**1b**){Ag(P_4_)}_4_][SbF_6_]_4_ · CH_2_Cl_2_ (**11**) crystallizing in the monoclinic space group *P*2/*n* are obtained (Scheme 5). The cationic part of **11** consists of one unit of **1b** that is coordinated to four Ag^+^ cations bearing a P_4_ tetrahedron as an η^2^‐coordinating ligand each (type **B**, Table 1), thus representing a unique molecular complex of a tetra‐coordinated *cyclo*‐P_5_ building block with a remarkably high content of P_4_.^[^ ^16^, ^17^, ^18^, ^19^, ^20^, ^21^, ^22^, ^23^, ^24^, ^25^, ^26^, ^27^, ^28^, ^32^ ^]^ Additional π‐interaction between the phenyl groups of the Cp^PEt^ ligand completes the distorted tetrahedral environment of the Ag^+^ cations and leads to a deviation of Ag^+^ from the *cyclo*‐P_5_ plane. In the solid state, two units of **11** are stacked with slightly slipped but almost parallel *cyclo*‐P_5_ ligands at an interplanar distance of 3.87 Å that suggests some stabilization via weak π–π interactions (Figure 2b). Five [SbF_6_]^−^ anions occupy the concave cavities between the [AgP_4_] units of the two complexes of **11**. Interestingly, the cavity at the free coordination site at the *cyclo*‐P_5_ ligand is only screened by a solvate molecule of CH_2_Cl_2_ (Figure S35), but all attempts to increase the amount of the [AgP_4_] units to a maximum of five by using a higher excess of AgSbF_6_ and P_4_ in the reaction failed.

**Figure 3:**
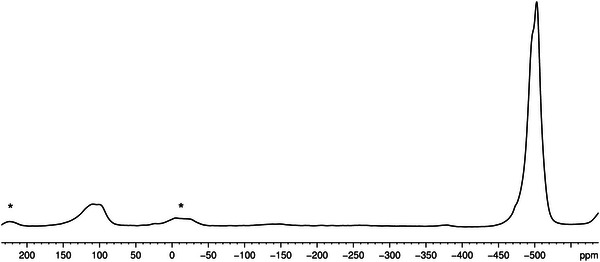
^31^P{^1^H} MAS NMR spectrum of **11**. Rotational sidebands are marked with asterisks.

The ^31^P{^1^H} MAS NMR spectrum of crystalline **11** displays a broad signal with a shoulder at −502 ppm for the intact coordinated P_4_ tetrahedra (Figure 3). The appearance of this shoulder can be explained by the position of the P_4_ units in the solid‐state structure of **11** leading to a chemically slightly different environment of the P_4_ units (cf. Figure S35). Furthermore, a broad signal with a shoulder at 103 ppm is observed corresponding to the different coordination modes of the P atoms of the *cyclo*‐P_5_ ligand.

To increase the amount of the [AgP_4_] units in compound **11**, *ortho*‐dichlorobenzene was used as a solvent instead of CH_2_Cl_2_ as in the case of compound **6** and in the expectation that larger solvent molecule will not fit into the cavity suited for the fifth silver cation. After stirring a 1:5:10 mixture of **1b**, AgSbF_6_, and P_4_ and layering it with *n*‐pentane, brown plates of **12** crystallize in the monoclinic space group *P*2_1_/*n* (Scheme 5). The asymmetric unit consists of [(**1b**)_2_(Ag_6.25_(P_4_)_8_)Ag_0.75_][SbF_6_]_7_ · 2 C_6_H_4_Cl_2_ (**12a**) with overall 10 Ag positions. In an idealized structure with all Ag positions fully occupied, a tetrameric structure would be built up by two pairs of opposed units of **1b** connected via silver cations. In fact, six Ag positions are fully occupied, while the remaining Ag^+^ cation is disordered over four positions (Figure S41) leading to different isomeric structures (see Supporting Information). Since other potential positions for the Ag^+^ cations are geometrically available in the crystal structure of **12**, it is not surprising that also crystals of [(**1b**)_2_(Ag_6.265_(P_4_)_8_)Ag_0.735_][SbF_6_]_7_ · 1.5 C_6_H_4_Cl_2_ (**12b**) with the same composition but with another distribution of the Ag^+^ cations were found in the course of our multiple attempts to obtain high‐quality single‐crystal X‐ray data (see Supporting Information). In **12**, the Ag─P(P_4_) bond lengths of the fully occupied Ag^+^ cations as well as the P─P distances of the P_4_ units agree with those found in **6c**, **11**, and in literature (Table 1).^[^ ^14^, ^57^, ^58^ ^]^

**Figure 4:**
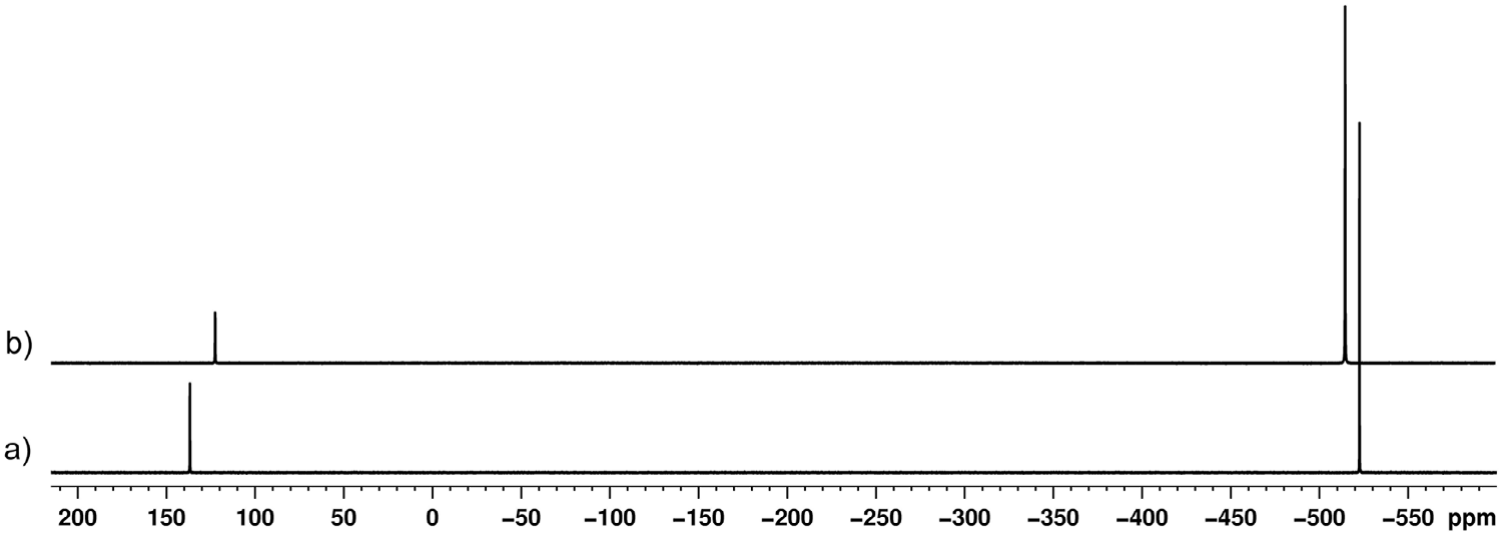
^31^P{^1^H} NMR of a) crystals of **11** in CD_2_Cl_2_/CD_3_CN and b) reaction solution of **11** in CD_2_Cl_2_.

*Using mixed‐ligand complexes* ***11*** *and* ***12*** *for the release of P_4_*. Since **11** is insoluble in common solvents and slightly soluble in CH_2_Cl_2_, it is convenient to use donor solvents to release the weakly bound P_4_ molecules. Crystals of **11** and **12** were redissolved in a mixture of CD_2_Cl_2_/CD_3_CN where partial decomplexation occurs in both cases. The ^31^P{^1^H} NMR spectrum of such solutions for **11** shows two sharp singlets at 136.8 ppm and −522.6 ppm corresponding to **1b** coordinated to Ag^+^ cations and to P_4_, respectively (Figure 4a). The shift of the latter signal corresponds to the one of free P_4_ (*δ *= −522 ppm). For comparison, the ^31^P{^1^H} NMR spectrum of the reaction solution in CD_2_Cl_2_ shows two singlets at 122.4 and −515.0 ppm (Figure 4b). Here, the signals for both the *cyclo*‐P_5_ ligand and the P_4_ molecules are shifted compared to the free compounds, indicating a coordination of both polyphosphorus ligands to Ag^+^ cations. Interestingly, singlets at −513 ppm and −515 ppm were observed for [P_4_Ag(GaCl_4_)]*_n_* and [Ag(η^2^‐P_4_)][Al(OC(CH_3_)(CF_3_)_2_)_4_], respectively, in their ^31^P{^1^H} NMR spectra in CD_2_Cl_2_.^[^ ^20^, ^22^ ^]^ This suggests that [Ag(η^2^‐P_4_)]^+^ units are present in the reaction solution.

The ^31^P{^1^H} NMR spectrum of **12** redissolved in a mixture of CD_2_Cl_2_ and CD_3_CN shows singlets at 151.9 and −520.7 ppm for the *cyclo*‐P_5_ moiety and free P_4_ units, respectively, for which the former signal is slightly shifted in comparison to the free ligand **1b**.

The partial decomplexation of compounds **11** or **12** in solutions of CH_2_Cl_2_/CH_3_CN can also be observed in the ESI‐MS spectrum in which the highest peak corresponding to [(**1b**)Ag]^+^ is centered at an *m*/*z* ratio of 905.06 (**11**) or 905.07 Da (**12**). Although the same crystals **11** used for the NMR study in solution were dissolved for the ESI‐MS measurement in CH_2_Cl_2_/CH_3_CN, no fragments containing P_4_ were detected, which is most probably due to the nonpolar nature of P_4_, which does not ionize under the conditions of ESI‐MS. No P_4_‐containing fragments were detected also for **12**.

## Conclusion

The comprehensive study of the reactions of the pentaphosphaferrocenes **1a** or **1b** with coinage M(I) salts (M = Cu, Ag) of WCAs in the presence of white phosphorus sheds light onto self‐assembly processes that lead to coordination rather than to encapsulation of tetrahedral P_4_ molecules (cf. Scheme 1). By variation of the stoichiometry in the two‐component self‐assembly reactions of [(Cp^PEt^Fe(η^5^‐P_5_)] (**1b**) with [Cu(CH_3_CN)_4_][SbF_6_], different coordination products were obtained. Besides the dimeric compounds **2** and **3**, the pentanuclear [(**1b**)Cu_5_(CH_3_CN)_15_][SbF_6_]_5 _· CH_2_Cl_2_ (**4**) was isolated, which represents the first discrete monomeric complex of a penta‐coordinated *cyclo*‐P_5_ building block that could be used as a precursor for efficient P_4_ coordination. However, such attempts proved fruitless due to competition between acetonitrile ligands and the weak donor P_4_, as well as the attempts to use acetonitrile‐free copper source. To replace Cu(I) by more Lewis acidic Ag(I) offered better perspectives since AgSbF_6_ is available in coordinated ligand‐free form. The two‐component self‐assembly reactions of [(Cp^R^Fe(η^5^‐P_5_)] (Cp^R^ = Cp* (**1a**), Cp^PEt^ (**1b**)) with AgSbF_6_ in different stoichiometric ratios lead to a series of coordination compounds **5**, **7**–**9**. Compounds **5** and **8** exemplify weakly coordinating CH_2_Cl_2_ molecules at the Ag^+^ ions suggesting that they can easily be removed to give space for P_4_ in subsequent three‐component self‐assembly reactions. Indeed, the CH_2_Cl_2_ ligands are thus partly or completely replaced by P_4_ in case of the reactions of **1a** with AgSbF_6_ and P_4_ depending on the stoichiometry and the solvent used, leading to the novel 1D polymeric compounds [(**1a**)_2_{Ag_2_(η^2^‐P_4_)_m_(CH_2_Cl_2_)_1‐_ *_m_*}]*_n_* [SbF_6_]_2_ *_n_* (**6**; *m* = 0.75 (**6a**), 0.67 (**6b**), 1 (**6c**)). However, the derivatives of **1a** form polymeric structures that render them completely insoluble, and the content of the coordinated P_4_ molecules is not very high (max. one P_4_ per unit **1a**). In contrast, using **1b** with the larger steric demand of its Cp^PEt^ ligand hinders the coordination of one or more **1b** units to the same Ag(I) cation. Moreover, the silver cations are additionally stabilized only by weak interactions with the aromatic substituents at the Cp^PEt^ ligand. Therefore, if the weak donor P_4_ is present, the self‐assembly does not proceed to the formation of polymers like **7**–**8** and multiple coordination of P_4_ becomes beneficial. Thus, the way to discrete monomeric complexes [(**1b**){Ag(η^2^‐P_4_)}_4_][SbF_6_]_4_ · CH_2_Cl_2_ (**11**) carrying the unprecedented content of four intact P_4_ moieties as ligands is enabled. The formation of this first complex of a tetra‐coordinated *cyclo*‐P_5_ ligand shows that a spectator Cp^R^ ligand can not only drive the reaction toward a molecular product but can also enhance the solubility of the resulting P_4_‐rich assembly. If the reaction is performed in *ortho*‐dichlorobenzene instead of CH_2_Cl_2_, molecular complexes **12** with the general formula [(**1b**)_2_(Ag_7_(P_4_)_8_)][SbF_6_]_7_ · *n* C_6_H_4_Cl_2_ (*n* = 2, 1.5) with a more complex structure are built up with as high content of P_4_ tetrahedra as in **11**. The disorder of one Ag atom in **12a** (*n* = 2) over alternative positions leads to different forms of the cocrystallizing P_4_‐rich complexes. These forms differ by the coordination of P_4_ in multiple positions and suggest the possibility of further increase of the P_4_ content provided that all partly occupied positions available for P_4_ coordination are filled.

Compounds **6**, **11**, and **12** are the first examples of P_4_‐rich mixed P*_n_*‐ligand complexes of coinage metals, a groundbreaking new type of product in this chemistry, capable of P_4_ coordination with controlled decomplexation. NMR spectroscopy studies in solution reveal that it is possible to release P_4_ by the addition of CH_3_CN to a suspension of the crystals of **6**, **11**, or **12** in CH_2_Cl_2_ under fragmentation of the coordination compounds. Furthermore, it was proven that crystals of the polymeric complex **6a** show no decomposition in air at room temperature for weeks.

The access to different naked polyphosphorus ligands in a mix‐ligand molecular complex opens an unexplored area of coordination of small molecules to coinage metal ions bound to a *cyclo*‐P_5_ building block. The possibility of using the obtained products for storage and transport of the intact P_4_ molecules for subsequent reactions will be further investigated. Additionally, other small molecules, especially air‐ or light‐sensitive molecules such as As_4_, that are capable of coordinating to metal ions, come into the focus of research, including studies for storage purposes or additional reactions after the release of the coordinated molecules. Aside from this, the novel mixed ligand metal complexes of naked polyphosphorus ligands may act as promising precursors and create new avenues in P_4_ transformation chemistry, thereby offering fresh insights into related research fields.

## Supporting Information

The authors have cited additional references within the Supporting Information.^[^ ^1^, ^2^, ^3^, ^4^, ^5^, ^6^, ^7^, ^8^, ^9^, ^10^, ^11^, ^12^, ^13^, ^14^, ^15^, ^16^, ^17^, ^18^, ^19^, ^20^, ^21^, ^22^, ^23^, ^24^, ^25^, ^26^, ^27^, ^28^, ^29^, ^30^, ^31^, ^32^, ^33^, ^34^, ^35^, ^36^, ^37^, ^38^, ^39^, ^40^, ^41^, ^42^, ^43^, ^44^, ^45^, ^46^, ^47^, ^48^, ^49^, ^50^, ^51^, ^52^ ^]^

## Conflict of Interests

The authors declare no conflict of interest.

## Supporting information



Supporting Information

## Data Availability

The data that support the findings of this study are available in the Supporting Information of this article.
